# The long noncoding RNA HOXA11‐AS promotes lung adenocarcinoma proliferation and glycolysis via the microRNA‐148b‐3p/PKM2 axis

**DOI:** 10.1002/cam4.5103

**Published:** 2022-08-04

**Authors:** Wenkun Chen, Xuena Li, Bulin Du, Yan Cui, Yu Ma, Yaming Li

**Affiliations:** ^1^ Department of Nuclear Medicine The First Hospital of China Medical University Shenyang China

**Keywords:** aerobic glycolysis, HOXA11‐AS, lung adenocarcinoma, microRNA‐148b‐3p, pyruvate kinase M2

## Abstract

**Background:**

Lung cancer is the most common malignancy in the world and a growing number of researches have focused on its metabolic characteristics. Studies have shown that the long non‐coding RNA (lncRNA) HOXA11‐AS is aberrantly expressed in many tumors. However, the role of HOXA11‐AS in lung adenocarcinoma (LUAD) glycolysis and other energy metabolism pathways has not been characterized.

**Method:**

The mRNA levels of HOXA11‐AS, microRNA‐148b‐3p (miR‐148b‐3p), and pyruvate kinase M2 (PKM2) were detected using qRT‐PCR. The expression levels of proteins were measured using immunohistochemistry and western blot. The CCK‐8, EdU, and colony formation assays were used to assess proliferation. Glycolytic changes were assessed by measuring lactate production, ATP production, and ^18^F‐FDG uptake. Bioinformatics analysis and dual‐luciferase reporter assays were used to characterize the relationship between HOXA11‐AS, miR‐148b‐3p, and PKM2. Proliferation and glycolytic changes were analyzed in xenograft tumor experiments using Micro‐PET imaging after downregulation of HOXA11‐AS in vivo.

**Results:**

The expression of HOXA11‐AS was markedly increased in LUAD, and was strongly associated with a poor prognosis. In addition, HOXA11‐AS promoted proliferation and glycolysis in LUAD, and miR‐148b‐3p inhibited proliferation and glycolysis in LUAD. Mechanistically, HOXA11‐AS positively regulated PKM2 expression by binding to miR‐148b‐3p, thereby promoting LUAD proliferation and glycolysis. In addition, HOXA11‐AS inhibited LUAD xenograft growth and glycolysis via upregulation of miR‐148b‐3p expression and downregulation of PKM2 expression in vivo.

**Conclusions:**

These results showed that HOXA11‐AS enhanced LUAD proliferation and glycolysis via the miR‐148b‐3p/PKM2 axis. The findings in this paper expanded our understanding of the molecular mechanisms of LUAD tumorigenesis and glycolysis and showed that HOXA11‐AS could be useful as a diagnostic and prognostic marker for LUAD. ^18^F‐FDG PET/CT can be used to visually evaluate the therapeutic effect of targeting HOXA11‐AS.

## Graphical abstract

This paper elaborately explored the role of lncRNA HOXA11‐AS and miR‐148b‐3p in glycolysis and proliferation of lung adenocarcinoma, and demonstrated that the lncRNA HOXA11‐AS is involved in the regulation of PKM2, a key protein of glycolysis, through sponging miR‐148b‐3p, which in turn affects the proliferation and glycolysis of lung adenocarcinoma.

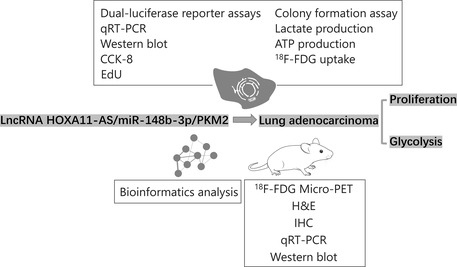

## 1 INTRODUCTION

Lung cancer is the most common malignancy in the world, accounting for 11.6% of all cancer cases. Lung cancer is the leading cause of cancer‐related deaths (18.4%).^1^ Lung cancer has been increasingly recognized as a serious worldwide public health concern. Lung cancer can be divided into two subtypes, non‐small cell lung cancer (NSCLC), which accounts for about 85% of all lung cancer cases,^2^ and small cell lung cancer. The incidence and mortality rate of lung adenocarcinoma (LUAD), the most common type of NSCLC,^3^ are increasing year by year.^4^

Despite improvements in strategies to treat NSCLC such as surgery, molecular targeted therapy, radiotherapy, and chemotherapy, the prognosis of most patients with NSCLC remains poor.^5^ Therefore, elucidation of the molecular mechanisms of lung cancer tumorigenesis and identification of molecular markers for early diagnosis of NSCLC is critical to early diagnosis, clinical treatment, and improved survival.

Metabolic reprogramming is an important feature of cancer that impacts proliferation, invasion, metastasis, and cancer survival.^6^ Tumor cells respond to metabolic stress and energy demands by reprogramming cellular metabolism, ultimately leading to tumor development and progression.^7^ Even under aerobic conditions, cancer cells select glycolysis over oxidative phosphorylation, a phenomenon called aerobic glycolysis. This phenomenon was discovered by Warburg in 1956 and is called the Warburg effect.^8^ A large amount of glucose is required in aerobic glycolysis to produce energy and support metabolic functions. Therefore, high aerobic glycolytic activity is an important indicator of cancer survival and growth.^9^ The pyruvate kinase M (PKM) gene is mainly present in tumors in the form of PKM2, a key glycolytic enzyme that can influence tumor glycolysis and a variety of tumor biological behaviors.^10^

Long non‐coding RNAs (lncRNAs) are a class of non‐coding RNA transcripts longer than 200 nucleotides that have limited or no protein‐coding funtion.^11^ Aberrantly expressed lncRNAs play a vital role in cancer proliferation, invasion, metastasis, cancer microenvironment formation, changes in metabolic function, and the immune response.^11^ Furthermore, lncRNAs can function as competing endogenous RNAs (ceRNAs).^12^ Long non‐coding RNAs act as sponges for miRNAs and bind to miRNAs through response elements to protect mRNAs from miRNA inhibition.^13^

A recent study showed that the lncRNA HOXA11‐AS was an important indicator of the development and progression of a wide range of human diseases.^14^ For example, HOXA11‐AS can act as an oncogene in multiple cancer types.^15^ In addition, HOXA11‐AS significantly impacts the development of LUAD. Increased expression of HOXA11‐AS in LUSC and LUAD was associated with proliferation, invasion, angiogenesis, and inhibition of apoptosis in NSCLC.^16^ A clinical study showed that Overexpression of HOXA11‐AS may indicate a poor prognosis in NSCLC.^17^ Previous studies have shown that HOXA11‐AS can function as a ceRNA in LUAD. In addition, HOXA11‐AS affects LUAD cisplatin resistance and can regulate the expression of STAT3 through sponging of microRNA‐454‐3p.^18^ Furthermore, HOXA11‐AS can promote NSCLC proliferation and inhibit apoptosis through the miRNA‐148a‐3p/DNA methyltransferase 1 (DNMT1) axis.^19^

These studies that HOXA11‐AS was associated with LUAD tumorigenesis and demonstrated that HOXA11‐AS may be a target for the treatment of LUAD. However, it is not clear whether HOXA11‐AS promotes LUAD proliferation through the modulation of glycolysis and its associated pathways. We investigated the role of HOXA11‐AS in LUAD glycolysis. We proposed that HOXA11‐AS may act as a molecular sponge for some miRNAs to influence the biological behavior of LUAD by regulating the expression of key proteins involved in glycolysis (e.g., PKM2). This study aimed to investigate the role and molecular mechanisms of HOXA11‐AS in glycolysis in LUAD and to identify novel therapeutic targets and strategies for lung adenocarcinoma.

## 2 MATERIALS AND METHODS

### 2.1 The Cancer Genome Atlas database and Kaplan–Meier Plotter

We measured HOXA11‐AS expression in normal lung tissues and LUAD. All data were obtained from the TCGA database. We selected the online database Kaplan–Meier Plotter^20^ to assess HOXA11‐AS prognostic value and used the JetSet best probe set to obtain Kaplan–Meier plots.

### 2.2 Cell culture

Human LUAD cell lines (A549, HCC827, H1299) and normal epithelial cells (HBE) were obtained from the Chinese Academy of Sciences Cell Bank.

### 2.3 Cell transfection

GenePharma (Shanghai, China) designed and constructed small interfering RNAs (siRNAs) that targeted HOXA11‐AS (si‐HOXA11‐AS), and negative control siRNAs (si‐NC). RiboBio (Guangzhou, China) designed and constructed miR‐148b‐3p mimics, microRNA mimic control (miR‐NC), miR‐148b‐3p inhibitor, and anti‐microRNA inhibitor control (anti‐miR‐NC). Overexpression vectors (HOXA11‐AS, PKM2) and empty vectors (Vector) were synthesized by SyngenTech. We used Lipo3000 (Thermo Fisher) for transfection according to the manufacturer's protocol. The siRNA sequences are listed in Table S1.

### 2.4 Western blot

Tissues and cells were lysed, and protein concentrations were measured using a BCA Kit (Beyotime). The proteins were separated by SDS‐PAGE, then transferred to polyvinylidene difluoride membranes, blocked, and incubated with primary antibodies. The primary antibodies and their dilutions were as follows: β‐Actin (1:2000), hexokinase‐II (HK2, 1:1000), glucose transporter type 1 (GLUT1, 1:100,000), PKM2 (1:1000), and lactate dehydrogenase A (LDHA, 1:2000) (Abcam). Proteins were detected using an ECL kit (Affinity). Images were acquired using a Bio‐Rad image analysis system (BIO‐RAD). Quantitative image processing was performed using ImageJ software (NIH).

### 2.5 Cell metabolic assays

After transfection for 48 h, metabolic status was determined using ^18^F‐FDG uptake, and ATP production and lactate production were measured using a γ‐counter, an ATP assay kit (Beyotime), and a lactic acid assay kit (KeyGen). One microcurie of ^18^F‐FDG was added per 1 ml of sugar‐free medium and incubated for 2 h in an incubator. The ^18^F‐FDG radioactivity in the supernatant and the cells was determined using a γ‐counter.

### 2.6 Dual‐luciferase reporter assay

The wild‐type and mutant reporter plasmids for HOXA11‐AS and PKM2 were synthesized by GenePharma for the luciferase reporter gene experiments. Transfection reagent was used to co‐transfect reporter plasmids and microRNA mimics negative control or miR‐148b‐3p mimics into cells. Luciferase activity was measured using a Dual‐Luciferase Reporter Assay (Promega).

### 2.7 Cell Proliferation, viability, and colony formation assays

Cell proliferation was determined using the CCK‐8 assay (Dojindo). After transfection, the cells were digested and counted, seeded into 96‐well plates at a density of 3000 cells per well, and incubated in a medium containing 10% CCK‐8 at four time points (0, 24, 48, and 72 h), then cultured for 2 h at 37°C in a 5% CO_2_ atmosphere. The absorbance was measured at 450 nm using a micro‐plate reader. To measure cell proliferation, we used the BeyoClick™ 5‐Ethynyl‐2′‐deoxyuridine (EdU)‐488 Assay Kit (Beyotime). After transfection, the cells were incubated with 10 μM EdU for 2 h, then fixed, permeabilized, and stained in a dark environment. The percentage of proliferating cells (stained by EdU, fluorescing green) compared to the total number of cells (stained by hoechst33342, fluorescing blue) was calculated using ImageJ software. Cell viability was determined using the colony formation assay. After transfection, cells were seeded at a density of 500 cells/well in six‐well plates, and cultured for 14 days at 37°C in a 5% CO_2_ atmosphere. After 14 days, the cells were fixed in 4% paraformaldehyde, then stained with crystal violet. Cells were counted and photographed using a digital camera.

### 2.8 RNA extraction and quantitative real‐time polymerase chain reaction

Total RNA was extracted using Trizol reagent (Takara), cDNA was reverse transcribed using PrimeScript™ RT Reagent Kit (Takara), and miR‐148b‐3p and U6 were reverse transcribed using Bugle‐Loop™ miRNA qPCR kit (RiboBio). Expression of RNA was detected using SYBR® PrimeScript™ RT‐PCR Kit (Takara, Japan). β‐actin was used as the internal reference for lncRNA and mRNA, and U6 was used as the internal reference for miRNA. Expression levels were quantitated using the 2^‐ΔΔCt^ method. RiboBio designed and synthesized all miRNA and U6 primers. ABM (Canada) synthesized all other primers used in this study. The primer sequences are listed in Table S1.

### 2.9 Mouse xenograft experiments and PET imaging

Twenty balb/c nude mice (5‐week‐old, female) were purchased from Huafukang and housed under SPF conditions. Tumor models were generated by subcutaneously injecting 2 × 10^7^ A549 or H1299 cells into the right dorsal side, and the rate of tumorigenesis was 70% in the end. Tumor volume was calculated using the following formula: tumor volume = length × (width)^2^ × 0.5. When tumor volumes reached approximately 300 mm^3^ the mice were randomly divided into two groups and intratumorally injected with si‐NC or si‐HOXA11‐AS for 2 weeks (5 nmol per mouse, 3 times per week). All mice underwent Micro‐PET (Madic) imaging following ^18^F‐FDG injection. Tumor tissues were isolated, weighed, and preserved for further experiments.

### 2.10 Histological examination

Tumor tissues were paraffin embedded and sectioned (5 μm). We used HE Staining Kit (Solarbio), UltraSensitive™ SP (mouse/rabbit) IHC Kit (MXB Biotechnologies), and DAB kit (MXB Biotechnologies) to perform HE and IHC staining. The antibodies and their dilutions were as follows: Ki67 (1:500), PKM2 (1:50), LDHA (1:200), HK2 (1:200), and GLUT1 (1:250) (Abcam). Tissue immunohistochemistry results were analyzed using the histochemistry score (*H*‐score). Histochemistry scores were calculated using the following formula: *H*‐score = ∑PI = ∑ (percentage score × intensity score). The intensity score was divided into four levels as follows: 0 (negative), 1 (weakly positive), 2 (moderately positive), and 3 (strongly positive). The percentage score was divided into the following four levels: 1 (0%–10% positive cells), 2 (11%–50% positive cells), 3 (51%–80% positive cells), and 4 (81%–100% positive cells).

### 2.11 Statistical analysis

Data are presented as the mean ± standard deviation (SD), and results were processed and statistically analyzed using SPSS 22.0 and GraphPad Prism 8.0.

Differences in groups were analyzed using Student's *t*‐test or one‐way ANOVA. *p < 0.05 was considered statistically significant.

## 3 RESULTS

### 3.1 HOXA11‐AS was overexpressed in LUAD and predicted poor prognosis

**FIGURE 1:**
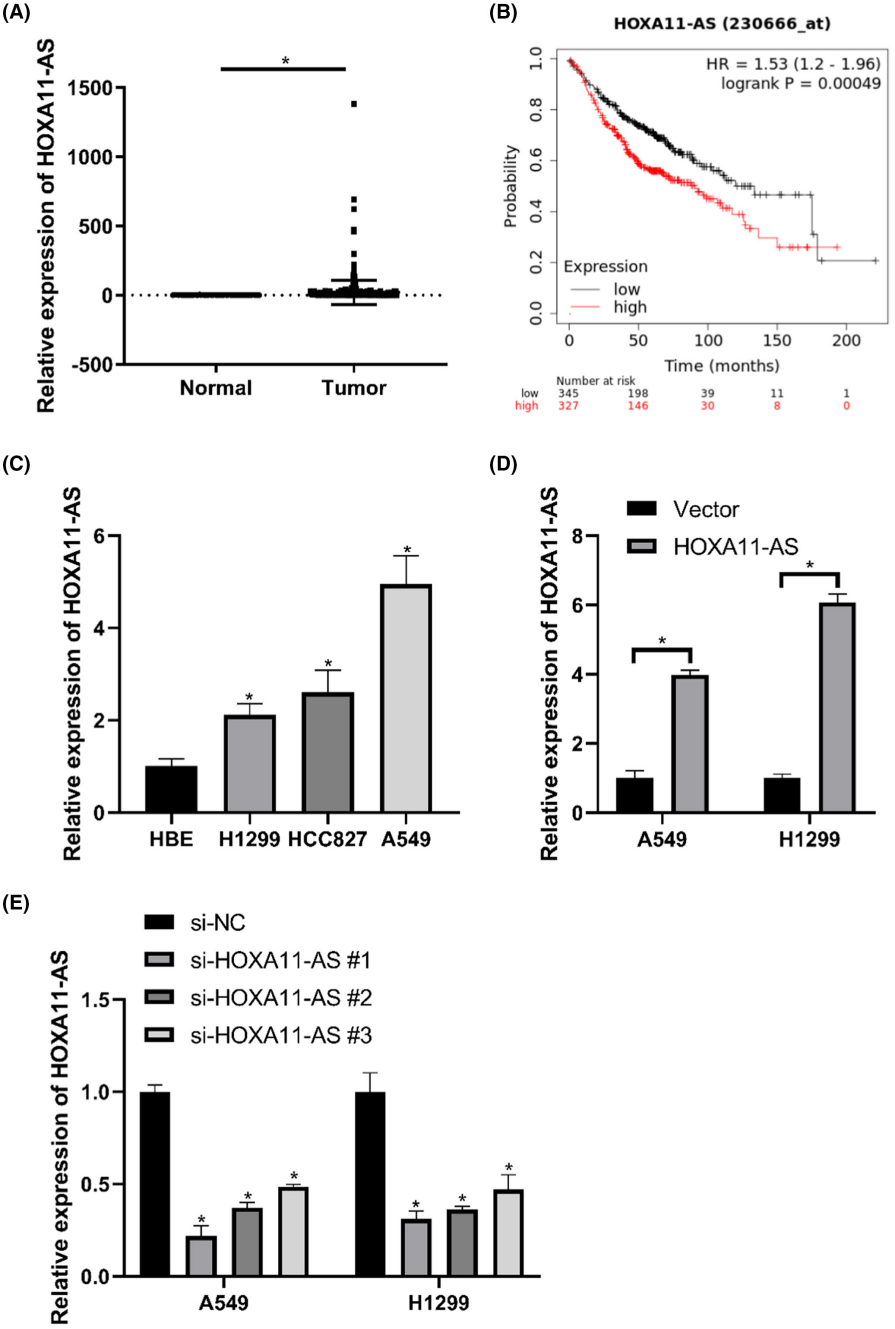
HOXA11‐AS was overexpressed in LUAD and predicted poor prognosis. (A) Analysis of HOXA11‐AS expression in LUAD tissues and normal tissues in TCGA database. (B) Survival analysis using Kaplan–Meier Plotter. The high expression group survival time was lower than that of the low expression group. (C) HOXA11‐AS expression in A549, HCC827, H1299, and HBE cells (qRT‐PCR). (D) HOXA11‐AS up‐regulation efficiency. (E) HOXA11‐AS down‐regulation efficiency. *p < 0.05.

We analyzed HOXA11‐AS expression in LUAD with data obtained from the TCGA database. The expression of HOXA11‐AS was higher in LUAD than that in normal lung tissues (Figure 1A). Kaplan–Meier Plotter was used to generate survival curves. The survival time of the high expression group was shorter than that of the low expression group (Figure 1B).

Quantitative RT‐PCR was used to evaluate the expression of HOXA11‐AS in cell lines. The expression of HOXA11‐AS was elevated in LUAD (Figure 1C). Notably, HOXA11‐AS expression was highest in A549 cells and lowest in H1299 cells. A549 and H1299 cells were chosen for further experiments. HOXA11‐AS was overexpressed or knocked down in A549 and H1299 cells using plasmids or siRNA. The expression of HOXA11‐AS was significantly increased in the overexpression group (HOXA11‐AS group) (Figure 1D) and decreased in the si‐HOXA11‐AS#1 group (Figure 1E) compared with that in the negative control (NC) group.

### 3.2 Overexpression or knockdown of HOXA11‐AS affected tumor proliferation and glycolysis in vitro

To further study the effects of HOXA11‐AS on LUAD proliferation and glycolysis, we examined changes in proliferation in LUAD cell lines after overexpression or downregulation of HOXA11‐AS using EdU, colony formation, and CCK‐8 assays. We also measured lactate production, ATP production, and ^18^F‐FDG uptake to characterize glycolytic changes.

**FIGURE 2:**
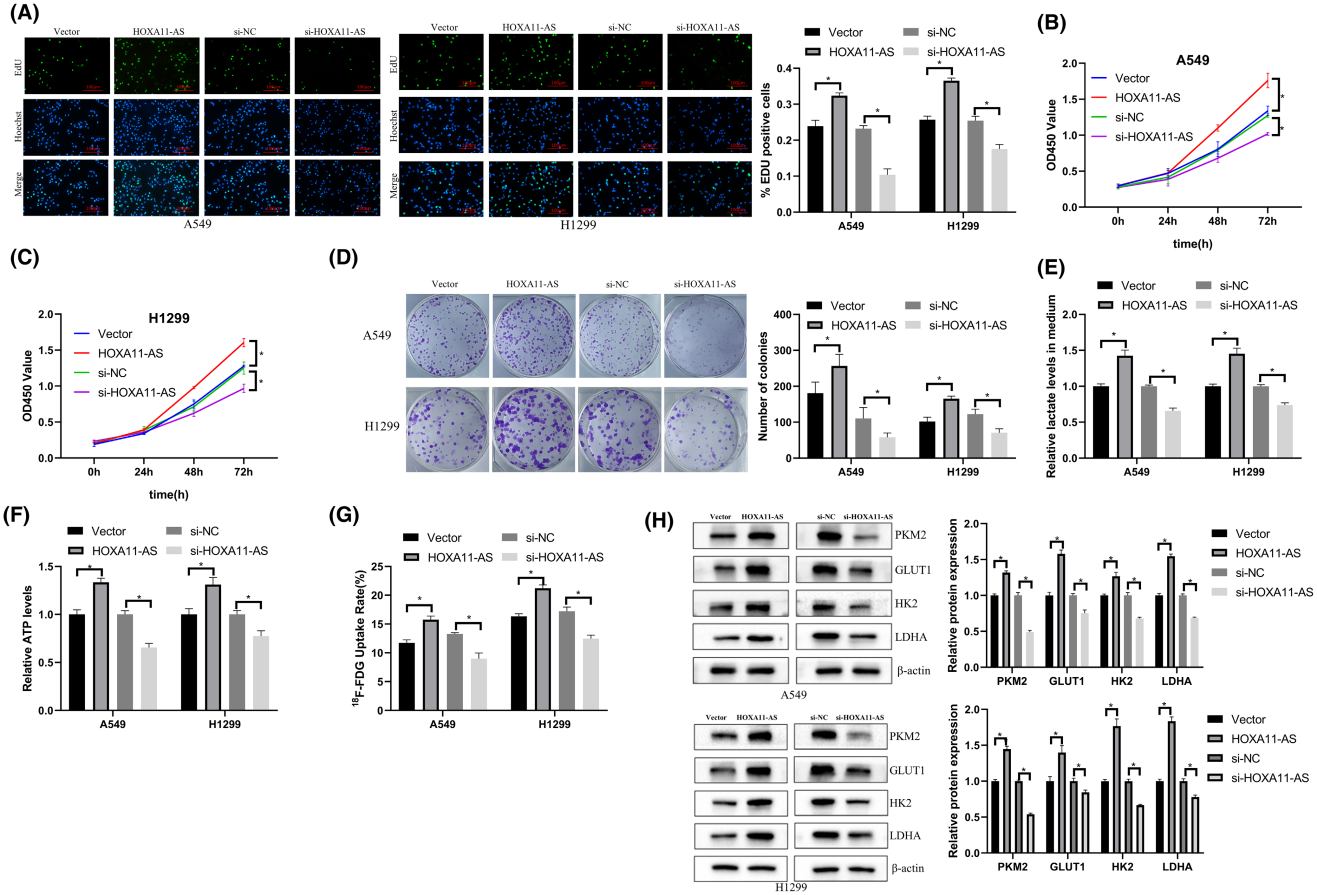
Overexpression or knockdown of HOXA11‐AS affected tumor proliferation and glycolysis in vitro. (A) EdU assay showed that HOXA11‐AS promoted proliferation (magnification 400×). (B and C) CCK‐8 assay. (D) Colony formation assay. (E) Lactate production assay indicated that HOXA11‐AS overexpression increased lactate production, and knockdown of HOXA11‐AS inhibited lactate production. (F) ATP production assay showed that ATP production increased after overexpression of HOXA11‐AS, and knockdown of HOXA11‐AS inhibited ATP production. (G) ^18^F‐FDG uptake assay showed that ^18^F‐FDG uptake increased after overexpression of HOXA11‐AS, and knockdown of HOXA11‐AS inhibited ^18^F‐FDG uptake. (H) Western blot showed that PKM2, GLUT1, HK2, and LDHA expression increased after overexpression of HOXA11‐AS, and knockdown of HOXA11‐AS inhibited PKM2, GLUT1, HK2, and LDHA expression. *p < 0.05.

The percentage of EdU‐positive cells in the HOXA11‐AS overexpression group was higher than that in the NC group and the percentage of EdU‐positive cells was significantly reduced after the downregulation of HOXA11‐AS (Figure 2A). The CCK‐8 assay showed that cell proliferation was increased in the high expression group, and was inhibited in the HOXA11‐AS downregulation group (Figure 2B,C). Downregulation of HOXA11‐AS significantly reduced cell colony formation, and overexpression increased the number of colonies (Figure 2D).

Knockdown of HOXA11‐AS significantly decreased ^18^F‐FDG uptake, lactate production, ATP production, and expression of glycolysis‐related proteins in cells. Overexpression of HOXA11‐AS increased ^18^F‐FDG uptake, lactate production, ATP production, and expression of glycolysis‐related proteins in cells (Figure 2E–H). These results showed that overexpression or knockdown of HOXA11‐AS affected proliferation and glycolysis in LUAD.

### 3.3 HOXA11‐AS acted as a ceRNA for miR‐148b‐3p

To further explore the mechanisms of HOXA11‐AS in LUAD progression, we used starBase^21^ to identify miRNAs that interact with HOXA11‐AS.

**FIGURE 3:**
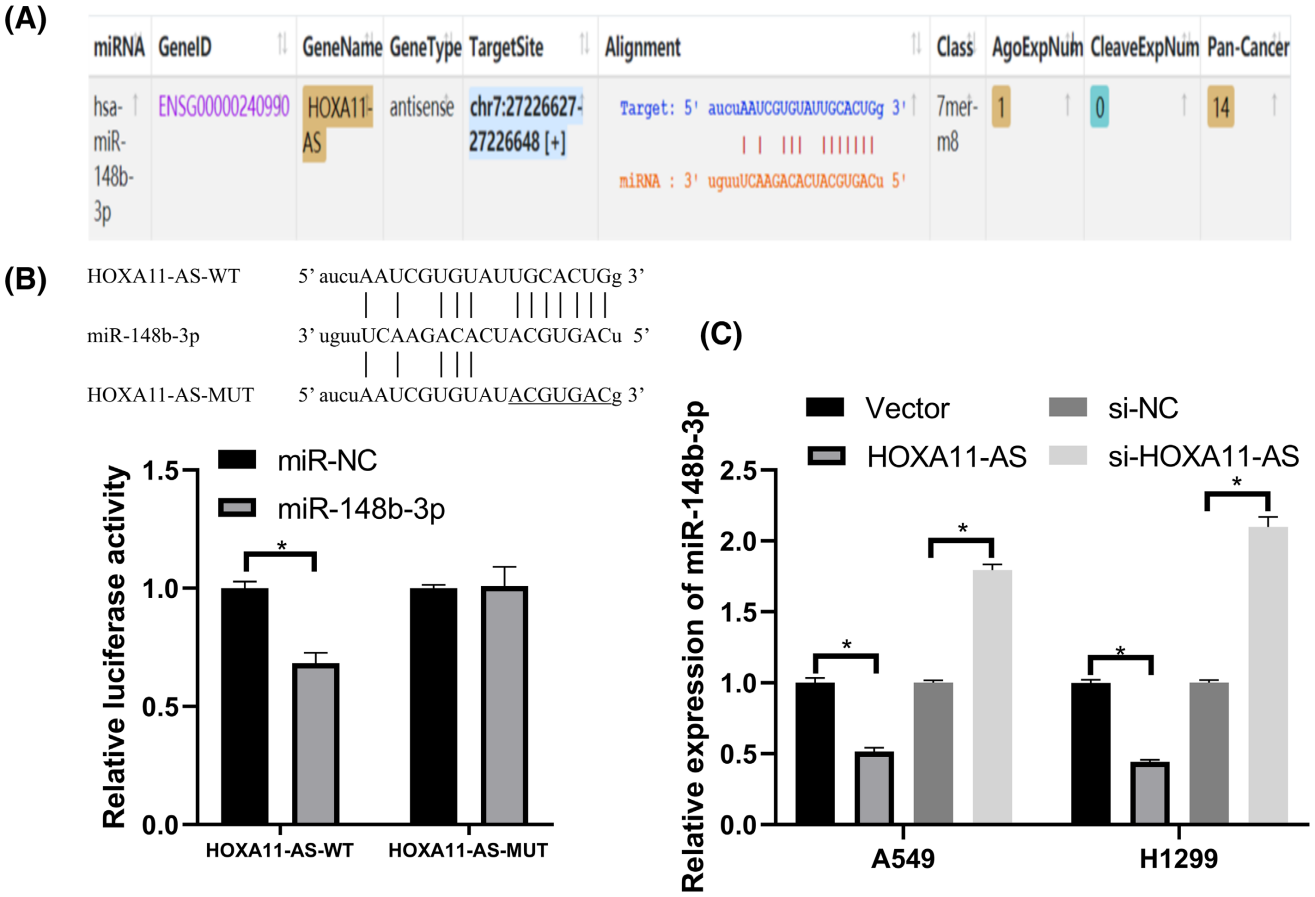
HOXA11‐AS acted as a ceRNA for miR‐148b‐3p. (A) The binding sites between HOXA11‐AS and miR‐148b‐3p predicted by starBase. (B) Luciferase activity detected after co‐transfection with miR‐148b‐3p mimic or miR‐NC and HOXA11‐AS‐WT or HOXA11‐AS‐MUT. (C) Overexpression of HOXA11‐AS resulted in decreased expression of miR‐148b‐3p, and knockdown of HOXA11‐AS resulted in increased miR‐148b‐3p expression. *p < 0.05.

We found that miR‐148b‐3p had a specific binding site for HOXA11‐AS (Figure 3A). We used the luciferase reporter gene assay to further verify whether HOXA11‐AS is bound to miR‐148b‐3p. The results showed that miR‐148b‐3p mimics reduced the luciferase activity in the HOXA11‐AS‐WT group compared with that in the HOXA11‐AS‐MUT group (Figure 3B). In addition, we further validated the interaction between HOXA11‐AS and miR‐148b‐3p using quantitative real‐time polymerase chain reaction (qRT‐PCR). The expression of miR‐148b‐3p was downregulated in response to HOXA11‐AS overexpression, and HOXA11‐AS knockdown resulted in increased expression of miR‐148b‐3p (Figure 3C).

These results showed that HOXA11‐AS and miR‐148b‐3p may act as a ceRNA regulatory system in which HOXA11‐AS acts as a competitive inhibitor of the binding of other mRNAs to miR‐148b‐3p.

### 3.4 MiR‐148b‐3p inhibited proliferation and glycolysis in LUAD

**FIGURE 4:**
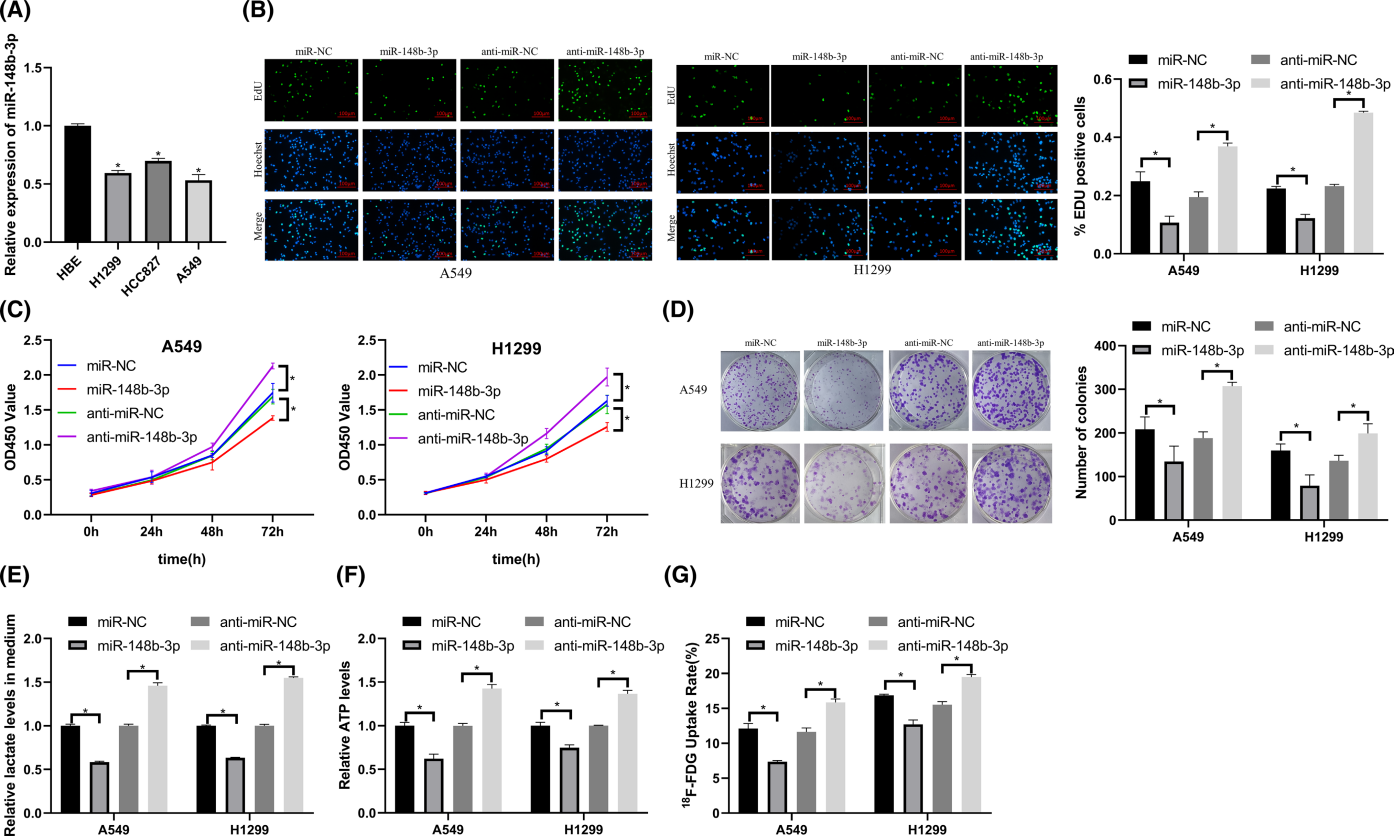
MiR‐148b‐3p inhibited proliferation and glycolysis in LUAD. (A) miR‐148b‐3p expression in A549, HCC827, H1299, and HBE cells (qRT‐PCR). (B) EdU assay showed that miR‐148b‐3p inhibited proliferation (magnification 400×). (C) CCK‐8 assay. (D) Colony formation assay. (E) Lactate production assay showed that miR‐148b‐3p overexpression reduced lactate production, and knockdown of miR‐148b‐3p promoted lactate production. (F) ATP production assay showed that ATP production was reduced after miR‐148b‐3p overexpression, and knockdown of miR‐148b‐3p promoted ATP production. (G) ^18^F‐FDG uptake assay showed that miR‐148b‐3p overexpression reduced ^18^F‐FDG uptake, and knockdown of miR‐148b‐3p promoted ^18^F‐FDG uptake. *p < 0.05.

We downregulation and overexpressed miR‐148b‐3p to determine its role in LUAD. We found that miR‐148b‐3p expression in LUAD cell lines (H1299, HCC827, A549) was lower than that in HBE cells (Figure 4A).

We transfected miR‐148b‐3p mimics and inhibitors into cells. After transfection, we evaluated LUAD cell proliferation and glycolysis using EdU, CCK‐8, colony formation, ATP production, lactate production, and ^18^F‐FDG uptake assays. Overexpression of miR‐148b‐3p inhibited LUAD proliferation and glycolysis, and downregulation of miR‐148b‐3p promoted proliferation and glycolysis in LUAD cells (Figure 4B–G).

These results showed that miRNA‐148b‐3p inhibited LUAD proliferation and glycolysis.

### 3.5 MiR‐148b‐3p targeted PKM2, a key enzyme in glycolysis

**FIGURE 5:**
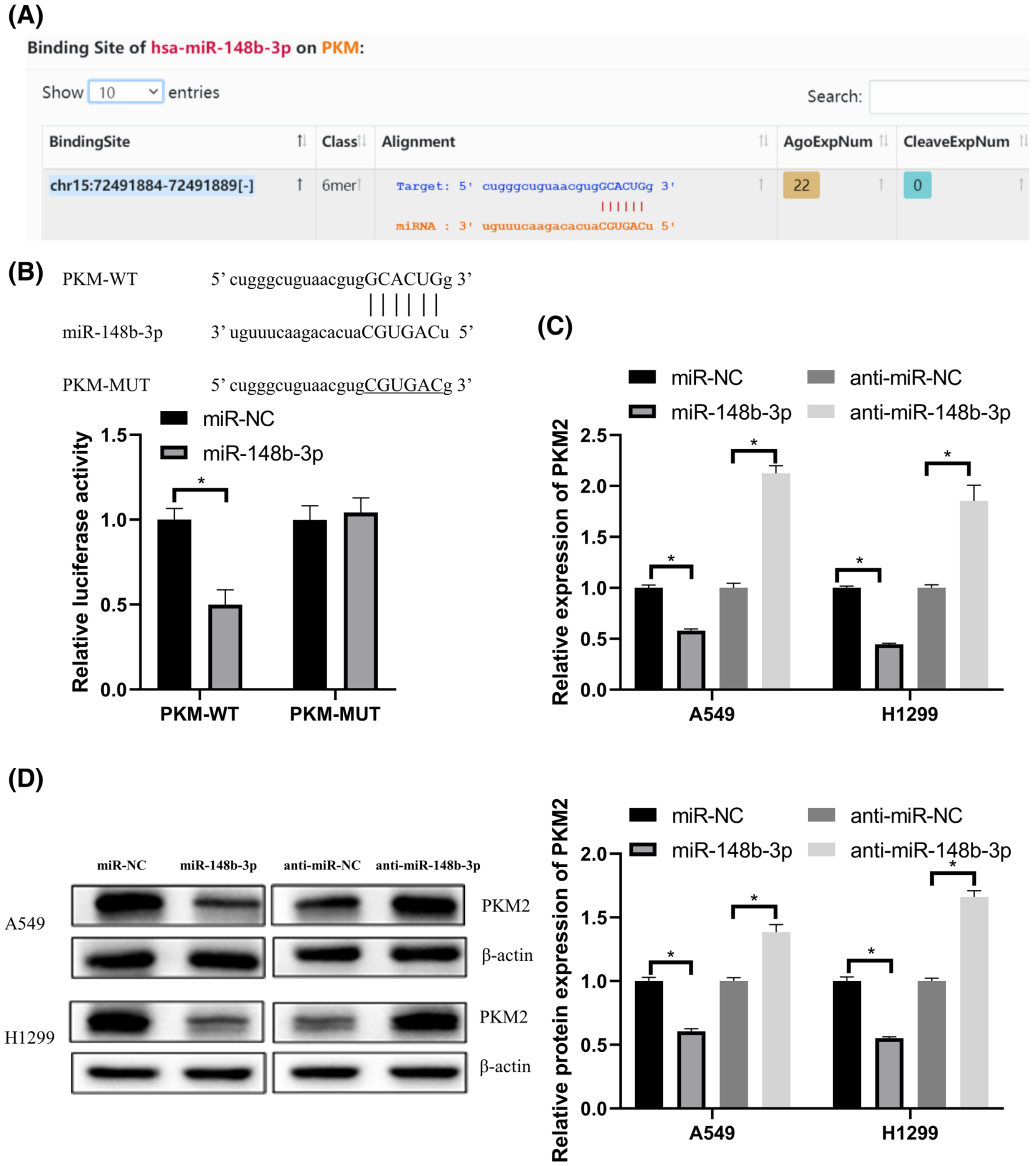
MiR‐148b‐3p targeted PKM2, a key enzyme in glycolysis. (A) Predicted PKM and miR‐148b‐3p binding sites by starBase. (B) Luciferase activity detected after co‐transfection with miR‐148b‐3p mimic or miR‐NC and PKM‐WT or PKM‐MUT. (C) qRT‐PCR showed that miR‐148b‐3p overexpression resulted in decreased PKM2 expression, and knockdown of miR‐148b‐3p resulted in increased PKM2 expression. (D) Western blot showed that miR‐148b‐3p overexpression resulted in decreased PKM2 expression, and knockdown of miR‐148b‐3p resulted in increased PKM2 expression. *p < 0.05.

We used starBase^21^ to predict the binding sequence between PKM2 and miR‐148b‐3p (Figure 5A). Luciferase reporter assay was used to verify that PKM2 was a target of miR‐148b‐3p (Figure 5B). qRT‐PCR analysis and western blot showed that PKM2 expression was suppressed by a miR‐148b‐3p mimic and increased by a miR‐148b‐3p inhibitor in LUAD (Figure 5C,D).

These data showed that miR‐148b‐3p regulated PKM2 expression by directly binding with PKM.

### 3.6 HOXA11‐AS regulated PKM2 through miR‐148b‐3p to promote proliferation and glycolysis in LUAD

We transfected si‐HOXA11‐AS, PKM2 overexpression plasmid, and miR‐148b‐3p inhibitor into cells to characterize the HOXA11‐AS/miR‐148b‐3p/PKM2 axis in LUAD cells.

**FIGURE 6:**
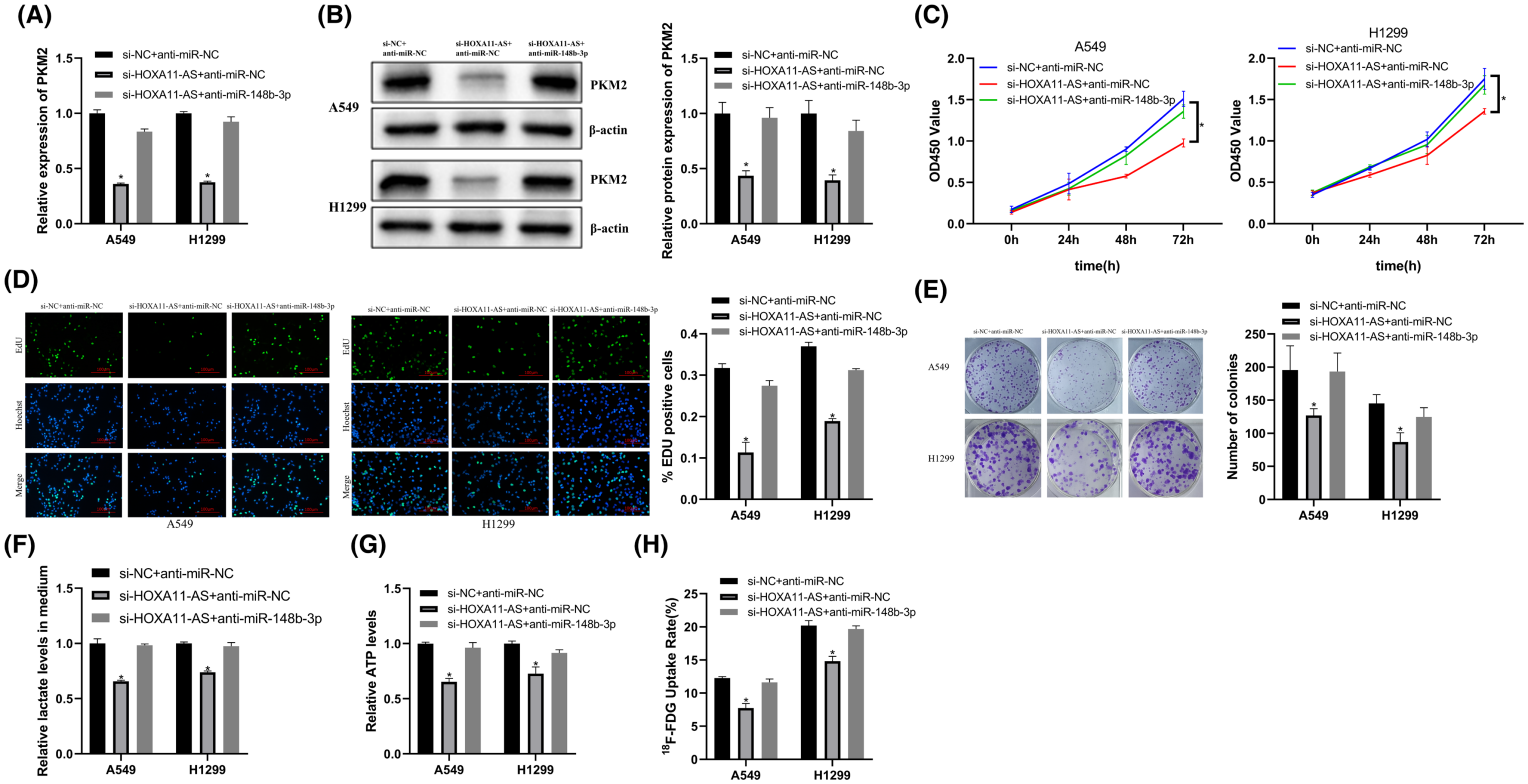
HOXA11‐AS regulated PKM2 through miR‐148b‐3p to promote proliferation and glycolysis in LUAD. (A) Down‐regulation of miR‐148b‐3p reversed PKM2 mRNA levels inhibited by si‐HOXA11‐AS. (B) Down‐regulation of miR‐148b‐3p reversed PKM2 protein levels inhibited by si‐HOXA11‐AS. (C) Down‐regulation of miR‐148b‐3p reversed the inhibitory effect on proliferation induced by si‐HOXA11‐AS (CCK‐8 assay). (D) EdU assay (magnification 400×). (E) Colony formation assay. (F) Down‐regulation of miR‐148b‐3p reversed si‐HOXA11‐AS‐induced inhibition of lactate production. (G) Down‐regulation of miR‐148b‐3p reversed si‐HOXA11‐AS‐induced inhibition of ATP production. (H) Down‐regulation of miR‐148b‐3p reversed si‐HOXA11‐AS‐induced inhibition of ^18^F‐FDG uptake. *p < 0.05.

We co‐transfected LUAD cells with si‐HOXA11‐AS and miR‐148b‐3p inhibitor. The results showed that miR‐148b‐3p inhibitor reversed HOXA11‐AS downregulation‐induced inhibition of PKM2 (Figure 6A,B) and also reversed the associated inhibition of glycolysis and proliferation of LUAD cells (Figure 6C–H). Then, we co‐transfected LUAD cells with si‐HOXA11‐AS and PKM2 overexpression plasmid. The results showed that PKM2 overexpression also reversed the HOXA11‐AS downregulation‐induced inhibition of PKM2 expression (Figure S1A,B), and reversed the associated inhibition of LUAD glycolysis and proliferation (Figure S1C–H).

These results showed that HOXA11‐AS regulated PKM2 via miR‐148b‐3p to affect LUAD proliferation and glycolysis.

### 3.7 Downregulation of HOXA11‐AS inhibited proliferation and glycolysis of LUAD xenografts in vivo via miR‐148b‐3p/PKM2

**FIGURE 7:**
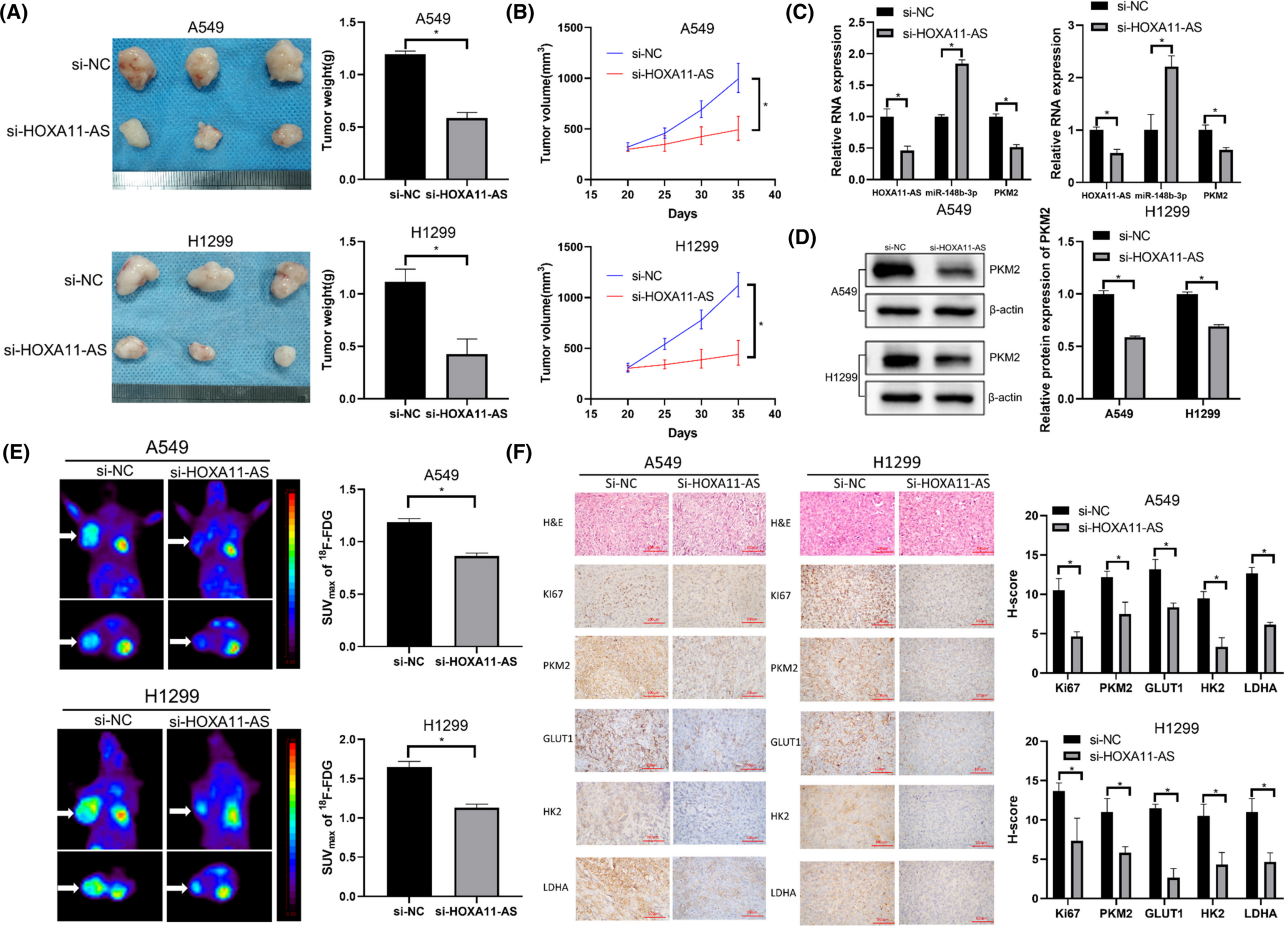
Downregulation of HOXA11‐AS inhibited proliferation and glycolysis of LUAD xenografts in vivo via miR‐148b‐3p/PKM2. (A) Picture and weights of tumor xenografts. (B) Tumor xenograft volumes. (C, D) The levels of HOXA11‐AS decreased, the levels of miR‐148b‐3p increased, and PKM2 mRNA and protein levels decreased in the si‐HOXA11‐AS group. (E) ^18^F‐FDG micro‐PET imaging of the si‐HOXA11‐AS and si‐NC groups. White arrows indicate the tumor xenografts. (F) Tissue sections from xenograft experiments were tested with H&E staining and IHC staining to detect Ki67, PKM2, GLUT1, HK2, and LDHA expression (magnification 400×). *p < 0.05.

We performed tumor xenograft experiments to investigate the biological effects of HOXA11‐AS. A549 and H1299 subcutaneous tumor xenograft models were constructed and HOXA11‐AS expression was downregulated. Tumor volumes and weights were significantly reduced in the si‐HOXA11‐AS group (Figure 7A,B). In the si‐HOXA11‐AS group, HOXA11‐AS expression was decreased, miR‐148b‐3p expression was increased, and PKM2 expression was decreased (Figure 7C,D). Micro‐PET imaging showed that tumor uptake of ^18^F‐FDG was lower in the si‐HOXA11‐AS group (Figure 7E). To evaluate whether HOXA11‐AS affected tumor proliferation and glycolysis, tumor tissue sections from the xenograft experiment were stained with Ki67, PKM2, LDHA, HK2, and GLUT1 antibodies. Staining intensity was lower in the si‐HOXA11‐AS group than that in the control group (Figure 7F). Therefore, downregulation of HOXA11‐AS inhibited LUAD proliferation and glycolysis in vivo via miR‐148b‐3p/PKM2.

## 4 DISCUSSION

Non‐small cell lung cancer accounts for the majority of lung cancer cases, and LUAD is the most common pathological type of NSCLC. Lung adenocarcinoma has a high mortality rate and poor prognosis.^1^ In addition, LUAD is associated with dysregulation of protein‐coding genes and non‐coding RNA gene networks.^22^

Tumor cells can construct a redox state and rapidly generate ATP by reprogramming metabolism, which promotes survival, growth, and metastasis. Metabolic reprogramming has long been recognized as a marker of cancer. Aerobic glycolysis is a key cancer marker and is important for tumor production, growth, and metastasis.^6^

Aerobic glycolysis can provide energy and supply the precursors and metabolic intermediates required for the synthesis of biological macromolecules to support cancer cell viability.^23^ Glycolysis can also result in increased lactic acid production, resulting in changes in pH in the tumor cell microenvironment. Changes in pH can affect the immune response, allowing tumor cells to evade immune detection.^24^ In addition, glycolysis can reduce ROS within tumor cells, resulting in increased cell stability. Glycolysis is the energetic basis for tumor onset and development. Studying the mechanisms of tumor glycolysis is vital to the understanding of tumorigenesis and the development of strategies to inhibit tumor development.^9^

Inhibition of the Warburg effect may be an effective strategy to treat LUAD. Many abnormally expressed lncRNAs have been identified in LUAD, providing novel targets for the study of LUAD pathogenesis. Studies have shown that lncRNAs are vital for the regulation of aerobic glycolysis in response to the energy demands of tumor cells.^25^ For example, LINC00930, an oncogenic lncRNA in nasopharyngeal carcinoma, promoted PFKFB3‐mediated glycolysis and histone modification, and targeting LINC00930 and PFKFB3 may be an effective approach to enhance radiosensitivity in patients with nasopharyngeal carcinoma.^26^ *Fusobacterium nucleatum* activated transcription of ENO1‐IT1 by increasing the binding efficiency of transcription factors to the promoter region of the lncRNA ENO1‐IT1. This increase in binding efficiency influenced the histone modification pattern of ENO1, resulting in increased glycolysis and tumorigenesis in colorectal cancer.^27^ The lncRNA glycoLINC can act as a backbone for phosphoglycerate kinase 1, phosphoglycerate mutase 1, ENO1, PKM2, and LDHA to enhance cellular glycolysis.^28^ These findings demonstrated that understanding the effects of lncRNAs on metabolism may allow for the characterization of metabolic regulatory mechanisms.

Our study showed that HOXA11‐AS promotes changes in proliferation and energy metabolism in LUAD, which provides a possible perspective for the study of HOXA11‐AS: HOXA11‐AS is associated with glycolysis‐related pathways. The expression of HOXA11‐AS was high in LUAD cell lines. We have fully demonstrated the effect of HOXA11‐AS on LUAD glycolysis and related gene expression in vitro by overexpressing and downregulation of HOXA11‐AS from two angles. Knockdown of HOXA11‐AS inhibited proliferation and glycolysis, and this change was reversed by miR‐148b‐3p inhibitors, which indicated that HOXA11‐AS could promote LUAD proliferation and energy metabolism through miR‐148b‐3p.

MicroRNAs play important roles in tumor development by targeting genes and regulating gene expression.^29^ We found that PKM2, a key enzyme in glycolysis, can bind with miR‐148b‐3p. Overexpression of PKM2 reversed the down‐regulation of HOXA11‐AS inhibition of LUAD proliferation and glycolysis. Downregulation of HOXA11‐AS suppressed PKM2 expression levels in vitro and in vivo. Studies have shown that PKM2 is a key glycolysis enzyme that regulates cellular metabolism through catalysis of the last glycolytic step, resulting in the conversion of phosphoenolpyruvate to pyruvate and the generation of ATP.^30^ In addition, PKM2 also can affect gene expression in the nucleus and can regulate cellular signaling pathways through phosphorylation.^30^

PKM2 expression is increased in tumors due to their highly proliferative properties and related metabolic requirements.^31^ In addition to acting as a signal modulator in the cytoplasm and a transcriptional regulator in the nucleus, PKM2 can also act as a transmitter of extracellular signals.^32^ PKM2 in the nucleus can influence gene transcription and cell cycle progression by binding β‐catenin, which ultimately promotes tumorigenesis.^33^ Many studies have shown that the knockdown of PKM2 inhibited cancer development.^31^ In addition, many studies have shown that PKM2 can directly affect tumor proliferation via various mechanisms.^30^, ^34^, ^35^

Previous studies have shown that lncRNA can influence the effects of PKM2 through multiple pathways. For example, NORAD sponges miR‐541‐3p and promotes PKM2 expression, which promotes prostate cancer bone metastasis through increased extracellular vesicle internalization.^36^ In addition, AC020978 binds and stabilizes PKM2, and promotes PKM2 intranuclear expression, thereby regulating HIF‐1α transcriptional activity.^37^ Nuclear LNCAROD can increase PKM2 expression in the nucleus via SRSF3‐mediated variable splicing of PKM, and cytoplasmic LNCAROD upregulates PKM2 expression via sponging miR‐145‐5p.^38^

In vivo conditions are more similar to the human environment than in vitro conditions, and the results of nude mice experiments could better reflect the role of HOXA11‐AS in humans. The application of Micro‐PET can build an imaging foundation for the future evaluation of the therapeutic effect of targeted therapy using ^18^F‐FDG PET/CT. Our further tumor xenograft experiments showed that knockdown of HOXA11‐AS reduced tumor volume and weight in vivo. Micro‐PET imaging showed reduced uptake of ^18^F‐FDG and inhibition of tumor glycolysis in the HOXA11‐AS downregulation group, a finding that was corroborated by subsequent immunohistochemistry results. Our study showed that HOXA11‐AS upregulated the expression of glycolysis‐related genes and increased enzyme activity to promote adaptation to metabolic changes in LUAD in vivo.

Our study was subject to some limitations. First, the role of HOXA11‐AS in LUAD was only evaluated in cell lines and nude mice, but not in human tissues. Therefore, the association between HOXA11‐AS expression and glycolysis in human LUAD tissues will be explored in the future. In addition, PKM2 can interact with many tumor‐related genes in the nucleus to influence tumorigenesis. Our study only explored the effect of HOXA11‐AS on PKM2 with regard to glycolysis, and other effects of modulation of PKM2 were not characterized. The detailed mechanisms of how HOXA11‐AS regulates or reprograms cellular energy metabolism to promote cancer cell proliferation, and whether these mechanisms affect LUAD invasion, metastasis, and apoptosis, have not been characterized. Further studies should investigate the mechanisms by which HOXA11‐AS regulates or reprograms cellular energy metabolism. In the future, we will explore the effects of HOXA11‐AS on various aspects of LUAD by affecting PKM2, and whether the findings could be extended to other lung cancer types. In addition, we will explore the possibility of targeting HOXA11‐AS in the clinical with our collaborating hospitals.

In conclusion, we found that HOXA11‐AS downregulation inhibited proliferation and glycolysis in LUAD cells. Our study showed that HOXA11‐AS can be a molecular sponge of miR‐148b‐3p, and the effects of HOXA11‐AS on LUAD could be reversed by miR‐148b‐3p and PKM2. In addition, HOXA11‐AS regulated miR‐148b‐3p‐mediated PKM2 expression. Our study indicated that HOXA11‐AS may be a therapeutic target for the treatment of LUAD. Our in vivo studies using Micro‐PET in nude mice could provide an imaging foundation for the future evaluation of the therapeutic effect of targeted therapy using ^18^F‐FDG PET/CT.

## 5 CONCLUSION

In conclusion, the lncRNA HOXA11‐AS was upregulated in LUAD and promoted LUAD proliferation and glycolysis. In addition, HOXA11‐AS may be a prognostic biomarker for LUAD. Our study showed that HOXA11‐AS promoted proliferation and glycolysis in LUAD by positively regulating PKM2 expression via sponging of miR‐148b‐3p. Therefore, HOXA11‐AS may be a potential molecular target for the treatment of LUAD. ^18^F‐FDG PET/CT can be used to visually evaluate the therapeutic effect of targeting HOXA11‐AS.

## AUTHOR CONTRIBUTIONS

WC, XL, and YL conceived of and supervised the study; WC obtained the data and performed preliminary analyses; WC and YL wrote and revised the manuscript; BD, YC, and YM proofread the manuscript; all authors read and approved the final version of the manuscript.

## FUNDING INFORMATION

This work was supported by the National Natural Science Foundation of China (no. 81761148029, 81971652).

## CONFLICT OF INTEREST

None.

## ETHICS APPROVAL

All experiments were approved and supervised by the Animal Research Committee of China Medical University (ethics approval number: CMU2020327).

## Supporting information


Figure S1

Table S1

## Data Availability

The data that support the findings of this study are available from the corresponding author upon reasonable request.
