# Supplementary material for: The long noncoding RNA HOXA11‐AS promotes lung adenocarcinoma proliferation and glycolysis via the microRNA‐148b‐3p/PKM2 axis
Source: Cancer Med. 2022 Aug 4;12(4):4421–33. doi: 10.1002/cam4.5103 (PMC9972162; doi:10.1002/cam4.5103)
Supplement: Supplementary file 1 — Figure S1 [file CAM4-12-4421-s001.docx]

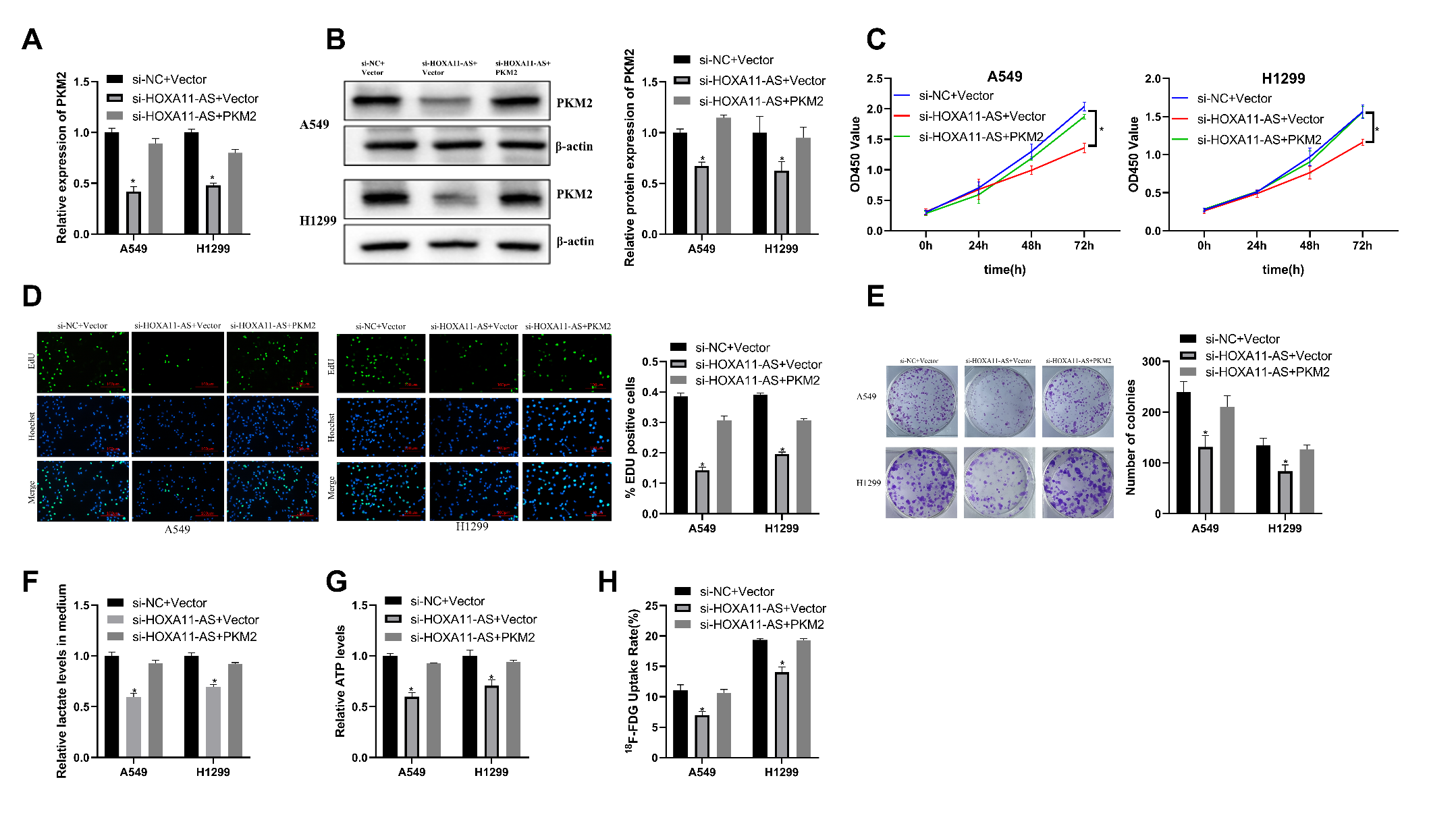


**Supplementary Material**

Fig S. HOXA11-AS regulated PKM2 through miR-148b-3p to promote proliferation and glycolysis in LUAD.

A, Overexpression of PKM2 reversed PKM2 mRNA levels inhibited by si-HOXA11-AS. B, Overexpression of PKM2 reversed PKM2 protein levels inhibited by si-HOXA11-AS. C, Overexpression of PKM2 reversed the inhibitory effect on proliferation induced by si-HOXA11-AS (CCK-8 assay). D, EdU assay (magnification 400×). E, Colony formation assay. F, Overexpression of PKM2 reversed si-HOXA11-AS-induced inhibition of lactate production. G, Overexpression of PKM2 reversed si-HOXA11-AS-induced inhibition of ATP production. H, Overexpression of PKM2 reversed si-HOXA11-AS-induced inhibition of ^18^F-FDG uptake. *, *P* < 0.05.
