# Supplementary material for: The long noncoding RNA HOXA11‐AS promotes lung adenocarcinoma proliferation and glycolysis via the microRNA‐148b‐3p/PKM2 axis
Source: Cancer Med. 2022 Aug 4;12(4):4421–33. doi: 10.1002/cam4.5103 (PMC9972162; doi:10.1002/cam4.5103)
Supplement: Supplementary file 2 — Table S1 [file CAM4-12-4421-s002.docx]

| Name | Sequences |
| --- | --- |
| β-actin primer | Forward：5’- GCGGGAAATCGTGCGTGACA-3’ |
|  | Reverse：5’- GGAAGGAAGGCTGGAAGAGTGC-3’ |
| HOXA11-AS primer | Forward：5’- AGCAACAGATCGTCACTCGG-3’ |
|  | Reverse：5’- GAGAACGAGGACCCTGCAAT-3’ |
| PKM2 primer | Forward：5’- ATGTCGAAGCCCCATAGTGAA-3’ |
|  | Reverse：5’- TGGGTGGTGAATCAATGTCCA-3’ |
| si-NC | Sense： 5’-UUCUCCGAACGUGUCACGUTT-3’ |
|  | Antisense：5’-ACGUGACACGUUCGGAGAATT-3’ |
| si-HOXA11-AS#1 | Sense： 5’-CCUGGUGGCUUGUCCGAUUTT-3’ |
|  | Antisense：5’-AAUCGGACAAGCCACCAGGTT-3’ |
| si-HOXA11-AS#2 | Sense： 5’-CCAACAGCCAGAACGGCUUTT-3’ |
|  | Antisense：5’-AAGCCGUUCUGGCUGUUGGTT-3’ |
| si-HOXA11-AS#3 | Sense： 5’-GCAAGCCUUGGGCUGCUUUTT-3’ |
|  | Antisense：5’-AAAGCAGCCCAAGGCUUGCTT-3’ |

Table S.

Primer and siRNA sequences used in this study.
